# Effect of Repeated Injection of Iodixanol on Renal Function in Healthy Wistar Rats Using Functional MRI

**DOI:** 10.1155/2018/7272485

**Published:** 2018-04-04

**Authors:** Yongfang Wang, Ke Ren, Lizhi Xie, Wenge Sun, Yi Liu, Songbai Li

**Affiliations:** ^1^Department of Radiology, First Affiliated Hospital of China Medical University, Shenyang 110001, China; ^2^GE Healthcare, MR Research China, Beijing, China

## Abstract

**Purpose:**

To determine the optimal time interval of repeated intravenous injections of iodixanol in rat model and to identify the injury location and causes of renal damage* in vivo*.

**Materials and Methods:**

Rats were randomly divided into Control group, Group 1 with one iodixanol injection, and Group 2 with two iodixanol injections. Group 2 was subdivided into 3 cohorts according to the interval between the first and second iodixanol injections as 1, 3, and 5 days, respectively. Blood oxygen level-dependent (BOLD) imaging and diffusion weighted imaging (DWI) were performed at 1 hour, 1 day, 3 days, 5 days, and 10 days after the application of solutions.

**Results:**

Compared with Group 1 (7.2%), Group 2 produced a remarkable R2^⁎^ increment at the inner stripe of the renal outer medulla by 15.37% (*P* = 0.012), 14.83% (*P* = 0.046), and 13.53% (*P* > 0.05), respectively, at 1 hour after repeated injection of iodixanol. The severity of BOLD MRI to detect renal hypoxia was consistent with the expression of HIF-1*α* and R2^⁎^ was well correlated with HIF-1*α* expression (*r* = 0.704). The acute tubular injury was associated with urinary NGAL and increased significantly at 1 day.

**Conclusions:**

Repetitive injection of iodixanol within a short time window can induce acute kidney injury, the impact of which on renal damage in rats disappears gradually 3–5 days after the injections.

## 1. Introduction

Contrast agents (CMs) have been largely applied in clinical applications, which are particularly required upon the performance of various radiographic imaging modes and complex interventional procedures [1]. Clinicians encounter risk factors that may lead to Iodinated Contrast-Induced Acute Kidney Injury (CIAKI) [2]. For instance, the patients with recurrent diseases may be required to undergo repetitive injections of contrast agents during examinations. There is a general agreement that the administration of multiple CMs within a short period of time may put the patients at risk [3]. The Contrast Media Safety Committee (CMSC) regards this as a serious matter in clinical practice, and it is of significance to propose the optimal time interval between the procedures that require intravascular CMs injections [2]. To our best knowledge, there was no previous publishing regarding this health risk.

Serum creatinine (sCr) has been used as a major diagnostic criterion for contrast-induced renal impairment, which typically reaches its peak at 48–72 h after contrast medium exposure [4]. This suggests an intrinsic delay of therapy in patients who may have developed CIAKI, while it prolongs the duration of hospitalization for patients without CIAKI [5]. Recent studies have been focusing on identifying the early diagnostic biomarkers of AKI [6]. Neutrophil gelatinase-associated lipocalin (NGAL) is proven to be one of the most significant biomarkers to detect CIAKI [7], which is observed to increase significantly prior to detectable changes in serum creatinine. Moreover, urinary NGAL (uNGAL) is demonstrated to reveal changes as early as 4 h after CM administration in rats [8] and 8 h in humans [9]. Therefore, uNGAL was applied as an indicator of renal injury after CMs in the present study.

The universally acknowledged causes of CIAKI include hypoxic and tubular damage as well as altered hemodynamics, despite the fact that not all the mechanisms leading to CIAKI have been revealed [10–12]. Renal hemodynamics and renal PO2 levels were investigated in Seeliger et al.'s study by using an optical probe and laser-Doppler fluxmetry, whereas the invasiveness of such method prevents its application on patients [13]. Quantitative functional MRI (fMRI) has attracted considerable attention in recent years as a noninvasive approach to assess relative renal content [14, 15]. BOLD MRI has been shown to be competent in providing indirect measurements of real-time oxygenation status for renal damage provoked by CM with deeper understanding [16, 17]. MR-based biomarker T2^*∗*^ is susceptible to changes in the volume of deoxygenated Hb (deoxyHb) per tissue volume element (voxel), and its reciprocal value (R2^*∗*^ = 1/T2^*∗*^) has been explored in numerous studies to evaluate tissue hypoxia. Pedersen et al.'s study verified a linear relationship between the measurements of R2^*∗*^ using BOLD imaging and the direct measurements of renal pO2 levels using oxygen sensitive microelectrodes in kidney [18]. However, recent report also showed that changes in R2^*∗*^ are potentially confounded by compartmental volumes (tubular, vascular, and interstitial compartments) [19]. In addition, diffusion weighted imaging (DWI) based on the molecular diffusion of water is feasible in the assessment of renal functions, particularly in the detection of early stage renal failure caused by CIAKI [20]. DWI can be applied to measure the magnitude of Brownian water motion; moreover, apparent diffusion coefficient (ADC) can quantitatively estimate water exchange rate between intra- and extracellular aspects of the kidney. Both techniques have been used extensively to assess the changes in renal physiology after CM injection. Preclinical studies were mostly conducted in a relatively short period of time, where little was published with regard to the changes of kidney function beyond 3 days after CM injection [8, 21]. It was recently revealed that CM can induce long-term damage of kidney in the model with acute kidney injury [22, 23]. Thereafter, BOLD and DWI MRI were performed to examine contrast-induced renal impairment in iodixanol-treated rats over a relatively longer duration of time (i.e., 10 days) after duplicated injection.

In order to achieve an optimal time interval that protects the renal system in rats, repeated intravenous injections of iodixanol with different time intervals were performed to induce aggravate kidney damage in healthy Wistar rats, which may also reveal differences in causing potential kidney injury with respect to time, location, and intensity of potential damage. The current study also aimed to examine if renal function can gradually return to a level close to baseline at a later time point. Meanwhile, the study allowed us to monitor kidney damage by assessing sCr and uNGAL to evaluate renal parenchymal injury at the cellular level using histopathology and to detect the cause of renal injury with immunohistochemistry.

## 2. Materials and Methods

### 2.1. Subject Selection

The current animal study was approved by the institutional ethics committee of China Medical University and performed complied with our institute's Guidelines for the Care and Use of Laboratory Animals. Wistar male rats 300 ± 20 g (*n* = 85) were randomly divided into Group 1 (*n* = 17) with one iodixanol injection, Group 2 (*n* = 51) with two injections, and the Control group (*n* = 17) with two saline injections separated by a 1-d interval. Group 2 was subdivided into 3 cohorts according to the interval between the first and second injections of iodixanol as 1-day, 3-day, and 5-day subgroups.

### 2.2. Experimental Protocols

**Figure 1:**
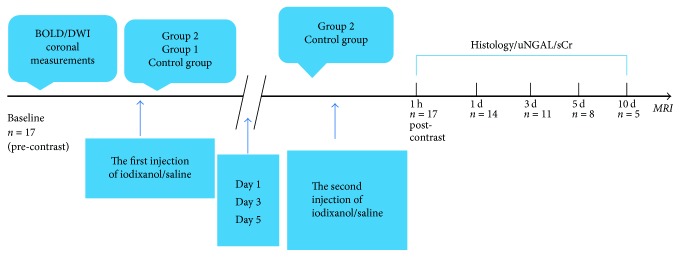
*The experimental flow chart*. This flowchart illustrates the specific measurement protocol and the time points of fMRI, histology, uNGAL, and sCr that occurred.

Rats were allocated in metabolic cages for urine collections. They had free access to food and water until 8 h before MRI examination. They were anesthetized via an intraperitoneal infusion dose of 10% chloral hydrate at 0.3 mL/100 g. Urine samples were collected prior to BOLD and DWI MRI acquisition. After baseline fMRI scan, contrast or physiological saline was inserted in the tail vein. Iodixanol (Visipaque 320, GE Healthcare, Ireland) was prewarmed (37°C) and uniformly injected as fast as possible. For Groups 1 and 2, intravenous contrast agent was administrated at a dosage of 4 g iodine/kg body weight. In Group 1, fMRI was performed at 1 h, 1 day, 3 days, 5 days, and 10 days after the injection of iodixanol. Group 2 underwent a second iodixanol injection at a respective interval of 1, 3, and 5 days after the first injection. The fMRI scan time points were 1 h, 1 day, 3 days, 5 days, and 10 days after the second application of iodixanol. After each fMRI acquisition, three rats were randomly selected and given an overdose of anesthetic; thereafter, the kidneys were immediately removed and the urine samples were collected at each respective time point. Physiological saline was used as placebo in place of iodixanol in Control group for the same scan (Figure 1).

### 2.3. MRI Protocol and Data Analysis

After 30 min of anesthesia, all MRI acquisitions were performed on a 3.0 T Twin Speed whole-body MR scanner (General Electric Medical Systems, Milwaukee, WI, USA) with a small extremity coil. In order to minimize the artifacts induced by bowel loops susceptibility, all the subjects were placed in the right decubitus position with their kidneys at the center of the rat array coil. BOLD and DWI images were independently acquired by 2 professional radiologists in a double-blinded manner. Data acquisition parameters are demonstrated in Table 1.

**Figure 2:**
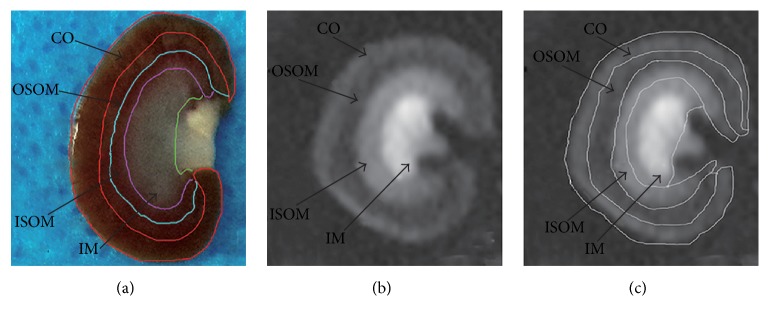
*The four kidney areas corresponding to anatomic image*. (a) Resected specimen. (b) T2 image. (c) The ROIs.

Parametric images of R2^*∗*^ and ADC were analyzed on ADVANCE 4.6 Workstation software (General Electric Medical Systems). According to the study of Li et al. [8, 24], all layers of renal tissues and the corresponding R2^*∗*^-weighted image were segmented into four regions, including cortex (CO), outer stripe of the outer medulla (OSOM), inner stripe of the outer medulla (ISOM), and inner medulla (IM) (Figures 2(a) and 2(b)). In each kidney, a single region of interest (ROI) corresponding to the histological sections was placed by a manual segmentation of the coronal BOLD images and DWI images. Moreover, the quantitative regional R2^*∗*^ and ADC measurements were performed. ROI was marked as large as possible (>18 mm^2^) (Figure 2(c)).

### 2.4. Pathology and Immunohistochemistry

In each group, three rats were sacrificed for histological studies at specific time points (1 h, 1 d, 3 d, 5 d, and 10 d). Fixation of kidneys with 4% paraformaldehyde for 72 h, dehydration, paraffin embedding, and sectioning (5-*μ*m) were performed for Hematoxylin-Eosin (H&E) staining. Two clinical pathologists with more than 5 years of experience independently examined the sections. At a magnification of 200x, intracytoplasmic vacuoles were revealed in the cortex, predominantly in the proximal convoluted tubules. Five different fields were randomly selected within each slide at each time point. The kidney was analyzed according to size and number of vacuoles, pronounced tubular dilatation, and interstitial vasodilation and congestion [25]. The severity of tubular injury was semiquantitatively analyzed with a scale of 0–4 assigned to each histopathological change, where 0 is no damage; 1, minimal injury (less than 5%); 2, moderate injury (between 5% and 25%); 3, intermediate injury (between 25% and 75%); and 4, severe injury (more than 75%) [26]. Hypoxia-inducible factor-1*α* staining could assess the degree of hypoxia in the intrarenal tissues for different groups. We evaluated renal parenchymal hypoxia semiquantitatively based on the degree of HIF-1*α* staining in the CO, OSOM, and ISOM, as well as IM. Grading was performed in a blinded manner. At 400x magnification, 5 randomly selected areas of each rat were scored according to both signal intensity and abundance [27], where 0 is no expression; 1, expression of <25% of the examination field; 2, expression of 26% to 50%; 3, expression of 51% to 75%; and 4, expression of >75% of the examination field; for signal intensity, 1 is moderate and 2 is strong.

Sections were pretreated in the same way as described above. Then, after deparaffinization, antigen retrieval, and peroxidase quenching, the samples were blocked with 5% normal goat serum and then incubated with anti-HIF-1*α* antibody (dilution 1 : 200) (Abcam, ab2185, Cambridge, MA, USA) overnight at 4°C. Subsequently, the tissue sections were incubated with the biotinylated anti-rabbit IgG secondary antibody (dilution 1 : 200) (A0277, Beyotime, Shanghai, China) for 1 h at room temperature.

### 2.5. Urinary Biomarker NGAL

In each group, after the injection of iodixanol or saline, 3 urine samples (0.5–2.0 mL) were collected at 1 h and 1, 3, 5, and 10 days. The samples were centrifuged at 3,200 rpm (4°C) for 20 minutes and subsequently placed into a −80°F freezer for storage. The concentration of urinary NGAL was analyzed using ELISA assays from Abcam (Cambridge, MA, USA), following the standard protocols. In order to minimize any confounding effects of urine flow rate, concentration levels of NGAL were normalized to urine creatinine concentrations (analyzed in local clinical laboratory) [28].

### 2.6. Serum Creatinine Assessment

To further verify the injury of kidney and the severity of the disease, blood serum creatinine was measured. The samples were collected from the venae angularis of the rats and were centrifuged at a speed of 3,500 rpm (4°C) for 20 minutes. The blood serum creatinine concentrations were analyzed in local clinical laboratory.

### 2.7. Statistical Analysis

SPSS22.0 (SPSS Inc., Chicago, IL, USA) was used for statistical analyses, where *P* value < 0.05 was considered as statistically significant. All the data were tested for normality. One way analysis of variance (ANOVA) was followed by LSD method or Kruskal-Wallis test (nonnormal distributions) to compare R2^*∗*^/ADC values across cohorts at the same time points. fMRI data in each group were compared with the baseline value using repeated ANOVA test or Bonferroni post hoc test. Spearman's correlation analysis was employed to assess the relationship between BOLD parameters and pathological variables.

## 3. Results

### 3.1. BOLD-Image Postprocessing Results

**Figure 3:**
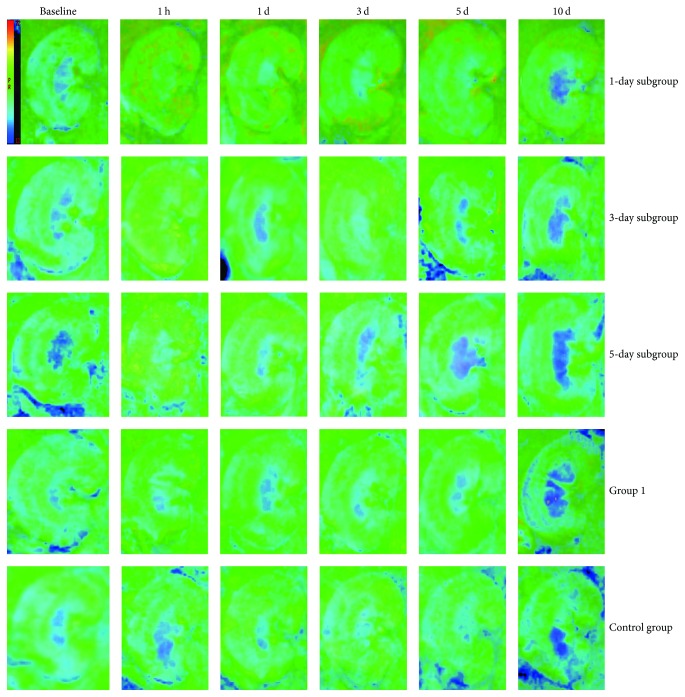
*Representative R*2^*∗*^* maps (baseline, 1 h, 1 day, 3 days, 5 days, and 10 days)*. All maps are demonstrated on the same window and level settings. 1-day, 3-day, and 5-day subgroups were injected for a second time at different time intervals. Group 1 was injected with iodixanol only once. The intensity of ISOM was larger than the remaining regions, which implies lower level of oxygenation.

The spatial resolution of R2^*∗*^ images was capable enough of distinguishing CO, OSOM, ISOM, and IM of the kidney (Figure 3). R2^*∗*^ values showed the least amount of changes in the Control group with respect to the time course, which confirmed the stability of BOLD MRI during the acquisition period.

**Figure 4:**
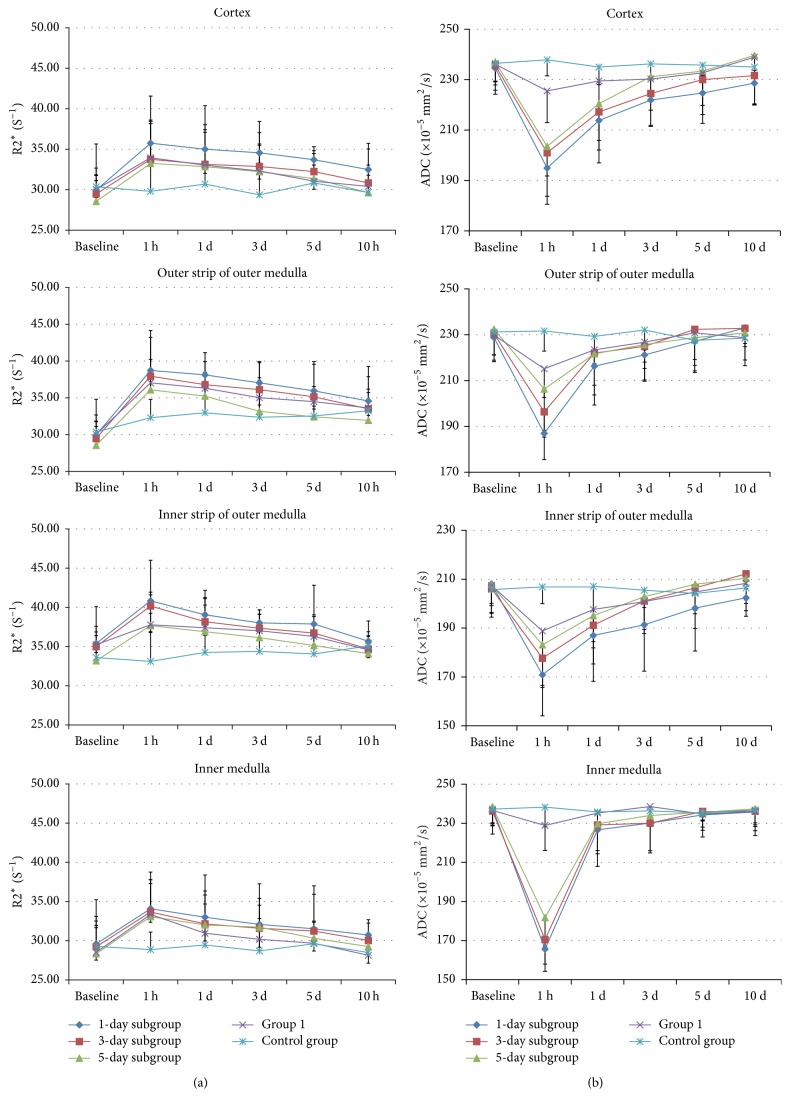
*Summary of the temporal changes in R*2^*∗*^*/ADC measurement during a long period of time*. (a) R2^*∗*^ time curves. (b) ADC time curves. Notice maximum R2^*∗*^/minimum ADC values appeared at 1 h, followed by gradual regression to a near-complete baseline level over time.

The montage image of R2^*∗*^ in the kidney of a representative rat was demonstrated in respect to time course over a 10-day period after iodixanol injection using the BOLD sequence. After repeated injection of iodixanol, R2^*∗*^ values increased significantly over time in the 1-day subgroup, where renal ISOM showed the largest differences in these tissues, and the duration of R2^*∗*^ (5 days) in ISOM was longer than that of other regions. ISOM demonstrated higher intensity of signals than that of CO in BOLD images, which probably indicated that oxygenation status was lower in ISOM. In OSOM, R2^*∗*^ values were more significant, while the duration (3 days) was shorter than that of ISOM. R2^*∗*^ values in IM showed a fast rise in the reduplicated-treated rats, followed by a return to baseline on day 3. In renal cortex, R2^*∗*^ values were continuously higher compared with baseline values at the time points (1 h to 5 days; all *P* < 0.05). A nearly complete recovery of baseline renal function was observed within 10 days in the 3-day subgroup, while Group 1 required 5 days to return to the baseline levels in all compartments.  Figure 4(a) presents that R2^*∗*^ values gradually recovered towards the baseline during the entire period of study in all 5 groups.

The average change of hypoxia levels in kidneys in each group was summarized in Table 2. Iodixanol increased R2^*∗*^ values to the maximum levels at 1 h, which was in accordance with previous animal studies [16, 29]. Compared with Group 1 (7.2%), Group 2 produced a remarkable R2^*∗*^ increment at inner stripe of the renal outer medulla by 15.37% (*P* = 0.012), 14.83% (*P* = 0.046), and 13.53% (*P* > 0.05), respectively, at 1 hour after repeated injection of iodixanol. These results indicated that short-term repeated injection of iodixanol is a risk factor for CIAKI, while 3–5-day interval is the optimal time interval for a second injection.

### 3.2. DWI-Image Postprocessing Results

**Figure 5:**
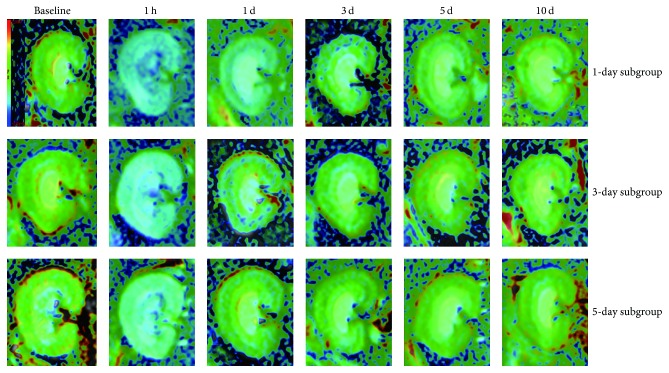
*Representative ADC maps (baseline, 1 h, 1 day, 3 days, 5 days, and 10 days)*. The cortical and medullary ADC in the kidney decreased in Group 2 after iodixanol injection, especially in 1-day subgroup. For each renal region, they significantly decreased at 1 h and thereafter gradually increased upward towards the baseline.

ADC values gradually recovered towards the baseline over time in all the groups (Figure 4(b)). In all the renal regions, ADC values showed a similar rapid initial decrease, reaching the bottom at 1 h after iodixanol administration, followed by a rebound towards the baseline (Figure 5). Among these 5 groups, the 1-day subgroup exhibited the greatest depression of ADC compared with the remaining groups. For each renal region (CO, OSOM, ISOM, and IM), a significant decrease in ADC lasted for 3 days in CO (*P* = 0.037) and OSOM (*P* = 0.005), as well as 5 days in ISOM (*P* = 0.008) in the 1-day subgroup. In IM, ADC values showed the strongest decline at 1 h (*P* = 0.003) but recovered to the baseline level after the first day. At the subsequent time points, ADC values in the 3-day subgroup gradually reached a level close to baseline at day 5 in all areas, while the 5-day subgroup recovered to the baseline level after 3 days. ADC values increased slightly for the rats injected with saline at 1 h, but they did not produce statistical significance for all the time points. At 1-hour time point, compared with that of Group 1, ADC values were significantly reduced by 1-day subgroup, 3-day subgroup, and 5-day subgroup in CO (all *P* < 0.001), OSOM (*P* < 0.001, *P* = 0.017, *P* > 0.05, resp.), ISOM (*P* = 0.038, *P* > 0.05, *P* > 0.05, resp.), and IM (all *P* < 0.001).  Table 3 summarized the time course for ADC values in Group 2.

### 3.3. Histologic Analysis

**Figure 6:**
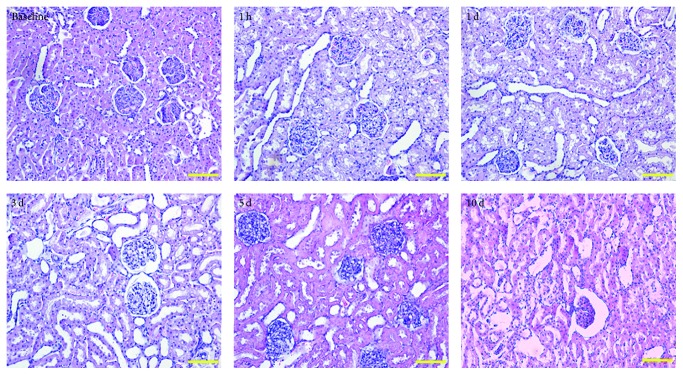
*Hematoxylin-Eosin (H&E) stained histological sections of the kidneys in the cortex*. Renal histological injury subjected to repeated injection of iodixanol at different time points in 1-day subgroup. Scale bar, 100 *μ*m.

A high incidence of vacuole formation occurred in all the subjects after the injection of iodixanol, especially in the repeated injection groups. In Group 2, the maximum modifications of vacuoles appeared at 1 h; on day 1, tubular epithelial cells swelled up and broke down; on day 3, pronounced tubular dilatation and mild vacuoles formation were observed after the intravenous repeated injection of iodixanol; on day 5, the glomeruli gradually became atrophy and fibrosis, and the proximal and distal convoluted tubule epithelial cells were cloudy and swollen; finally, on day 10, the glomeruli exhibited overt atrophy and fibrosis, and a small amount of interstitial vasodilation was revealed (Figure 6).

**Figure 7:**
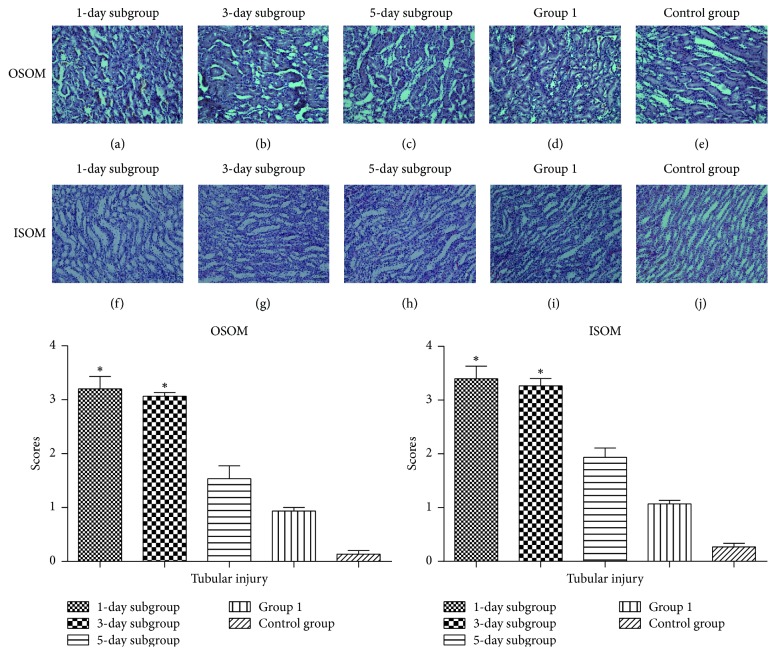
*Hematoxylin-Eosin (H&E) stained histological sections of the kidneys in the outer medulla (OSOM and ISOM) at 1 h in different groups*. Remarkable changes (swollen, broken down, necrotic, and intraluminal desquamation) were observed in part of proximal tubular and distal convoluted tubular epithelial cells in the OSOM and medullary thick ascending limbs, and medullary collecting ducts in the ISOM. Scale bar indicates 100 *μ*m. Asterisk indicates *P* < 0.05 (compared with Group 1).

At 1 hour, the magnitude and severity of renal tubular damage depended on the frequency and interval of injections. Compared with Group 1, the highest severity was observed after the repeated injection with a short interval of only 1–3 d in OSOM (*P* = 0.031, *P* = 0.049, resp.) and in ISOM (*P* = 0.031, *P* = 0.048, resp.); in addition, the least severe tubules injury was present after the treatment with iodixanol for both OSOM and ISOM in the 5-day subgroup (*P* > 0.05, *P* > 0.05, resp.) (Figure 7).

### 3.4. HIF-1*α* Immunohistochemistry Results

**Figure 8:**
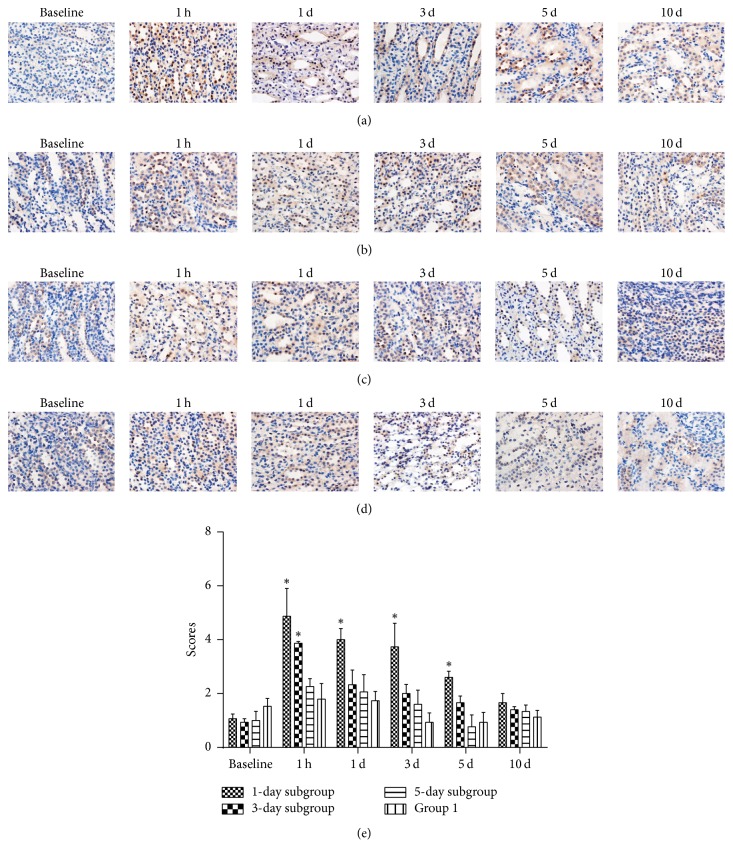
*HIF-1α signals changes in ISOM renal zones*. HIF-1*α* was mainly localized in the renal ISOM, especially at 1 h. Marked nuclear accumulation of HIF-1*α* appears in the 1-day subgroup. Thereafter, staining gradually decreased over time, and they were detectable for 5 days. Asterisk indicates *P* < 0.05 (compared with Group 1). Scale bar, 50 *μ*m. (a) 1-day subgroup; (b) 3-day subgroup; (c) 5-day subgroup; (d) Group 1; (e) HIF-1*α* score.

After repeated injection of iodixanol, hypoxia-inducible factor-1*α* (HIF-1*α*) was transiently upregulated at 1 h, the expression of which was most significant in ISOM area. HIF immunostaining was confined to a short period of time, within 5 days after the induction of hypoxic insult in Group 2 (Figure 8). 3-day and 5-day subgroups showed less expression of HIF-1*α* than 1-day subgroup at 1 hour. At 1 hour, compared with Group 1, immunostaining of renal HIF-1*α* was aggravated in Group 2 after the second injection of iodixanol in ISOM (*P* = 0.008, *P* = 0.044, *P* > 0.05, resp.).

### 3.5. Urinary Neutrophil Gelatinase-Associated Lipocalin

**Figure 9:**
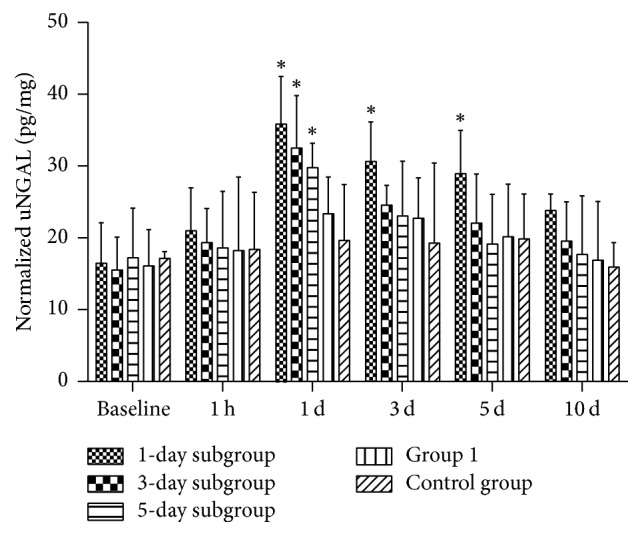
*The normalized uNGAL (pg/mg) at different time points during the course of contrast injection (n* = 3). There was a significant increase in uNGAL levels in rats at 1 day, 3 days, and 5 days in 1-day subgroup. Asterisk indicates *P* < 0.05 (compared with baseline).

CIAKI was confirmed to decrease in renal function after iodixanol injection using urinary neutrophil gelatinase-associated lipocalin (uNGAL) as a marker. As shown in Figure 9, the statistically significant change of uNGAL was observed after iodixanol injection within 5 days for Group 2.

### 3.6. Serum Creatinine Results

As shown in Table 4, the sCr concentration reached its maximum value on day 3 in 1-day subgroup and 3-day subgroup (*P* = 0.036, *P* = 0.035 versus baseline, resp.), whereas the difference was not statistically significant at other time points in all groups (*P* > 0.05 versus baseline).

### 3.7. Correlation between BOLD Parameters and Renal Injury and HIF-1*α* Expression Scores

**Figure 10:**
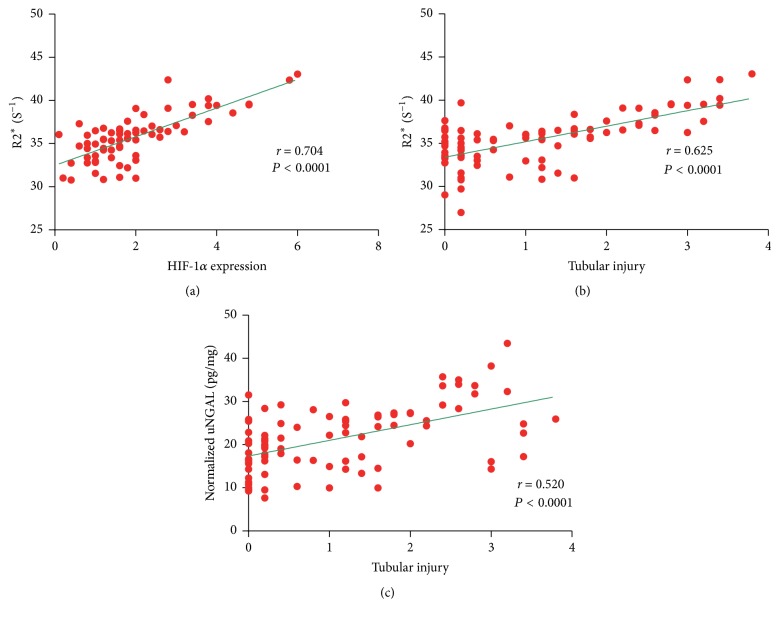
*Correlation of R*2^*∗*^* with HIF-1α expression/tubular injury and tubular injury with uNGAL*. (a) The correlation between the measured R2^*∗*^ values and HIF-1*α* expression score in the ISOM. (b) The correlation between the measured R2^*∗*^ values and tubular injury score in ISOM. (c) The correlation between all the measured uNGAL and tubular injury score in ISOM.

As shown in Figure 10, the correlation between BOLD parameters and pathological scores, HIF-1*α* expression scores of inner stripe of outer medulla, was decided by linear regression analysis. There was a good correlation between R2^*∗*^ and HIF-1*α* expression (*P* < 0.0001; *r* = 0.704); and R2^*∗*^ was found to be well correlated with pathological scores (*P* < 0.0001; *r* = 0.625). Meanwhile, there was fair correlation between histological scores and urine NGAL (*P* < 0.0001; *r* = 0.520).

## 4. Discussion

Plenty of guidelines on CIAKI proposed to avoid multiple injections with CMs on patients especially within 48–72 hours after the first injection [2, 30]. However, optimization of the clinical interval is of significance for the supervision of such treatments and prevention of the occurrence of CIAKI in clinical practice. Additionally, iodixanol has been widely observed in recent studies due to its iso-osmolality with plasma [31]. Therefore, the current study is a preliminary assessment to the impact of repetitive iodixanol injections on the damage sites in order to optimize the interval of repeated iodixanol administration.

The mechanisms leading to direct CIAKI have not been fully understood. However, the previously revealed potential risk factors act in a time and concentration dependent manner, indicating that the duplicate injection of CM in a short-term duration of time may aggravate acute damage of kidney [2]. In the current study, a detected increase in R2^*∗*^ after CM injections in Group 2 may be associated with the occurrence of CIAKI as determined by urinary NGAL. The escalated distribution of HIF-1*α* expression in renal tissues highlighted the degree of cellular hypoxia and a subsequent transcriptional response in CIAKI. Our results demonstrated a significant increased risk of CIAKI among the subjects that received a second dose of CM within one day after their first injection. Litter aggravate renal damage was observed in 3-day or 5-day subgroups. Moreover, a decreased expression of HIF-1*α* was observed in rats with the second injection on 3 or 5 days after their previous injection, which suggested a longer interval time between two CM injections. The possible mechanism may relate to cytotoxic effect, since iodixanol cytotoxicity is mostly (~85%) observed within 15 minutes, to the maximum at 3 hours [32]. Taken together, it was reported that 70–85% of the injected contrast agent was cleared within 24 hours in humans [33], which reinforced the statement that even a short period of repeated exposure could activate the cascade leading to kidney damage but mostly the cumulative effect of that initial exposure vanished in the later time periods (after 5 days).

There was a notable elevation of R2^*∗*^ at 1 h after the second injection of iodixanol observed among all renal areas in Group 2. In CIAKI-rats, BOLD MRI showed that ISOM demonstrated the most sensitive responses to repeated iodixanol administration. This was further confirmed by HIF-1*α* immunohistochemical staining findings. Previous studies on ISOM region revealed its sensitivity to ischemic injury [34, 35]. One possible explanation is that iodixanol diverts medullary blood flow to increase cortical blood flow and results in the deterioration of medullary perfusion and the reduction of renal oxygen tensions [36]. On the other hand, hypoxia was observed to correlate with tubular injury as well [37]. NGAL, as a viable tubular damage marker, is rapidly and massively generated in tubule cells of the kidney after renal ischemia reperfusion, including those of ISOM, released into the urine within hours [38]. Our findings showed that uNGAL was significantly detected within 5 days after the injection of iodixanol, indicating the existence of renal tubular acute injury. In addition, the data presented here are warranted to correlate the observed BOLD MRI changes with both HIF-1*α* expression and histopathological changes in the medulla. Therefore, an increase in R2^*∗*^ values in ISOM could contribute to determining the risk for subsequent development of CIAKI as evaluated by NGAL [39].

In the current study, the influence of iodixanol on renal water diffusion was evaluated dynamically during the 10 days after the second injection. The prominent decrease of ADC in iodixanol-treated rat groups is aligned with a previous study [40]. One possible explanation of such observation is that the vacuolization found in the cortex may have increased the ratio of cytotoxic edema. Secondly, there was a reduction of renal blood flow that could in return cause longer-lasting iodixanol retention. More importantly, the third contributor could be the changes of tubular fluid resulting from the influenced viscosity of iodixanol, which in return reduced glomerular filtration. Yet, several previous studies suggested a positive linear correlation between glomerular filtration (GFR) and the renal ADC [20, 41].

Similarly, this phenomenon of long-term renal injury caused by iodixanol was reported in clinical routine [42]. It was previously showed that some cases of CIAKI possessed a higher risk to develop a persistent renal damage which regressed to a level close to baseline after over 3 months of time [43]. It was also indicated that AKI might have increased the risk for chronic kidney disease and end-stage renal disease [44]. Thus, CIAKI is not a transient, but a direct cause of aggravated renal functions [45]. Nevertheless, our results confirmed the existence of correlations between CIAKI and longer-lasting renal hypoxia indicated by BOLD findings following repetitive CM injections. The reason may due to the fact that kidney damage caused by the first injection made the subjects more vulnerable to acute kidney injury that could be induced by further injections. Our observations were consistent with clinical report that repetitive iodixanol injections faced higher-risk recurrent episodes of kidney injury and a long-term loss of kidney function. It was shown that one-third of patients with AKI during their initial hospitalization experienced repeated episodes of AKI, while each AKI episode doubled the danger of progressive chronic kidney disease [46].

In this study, a comprehensive method used sCr and uNGAL to evaluate renal damage after CM repeated injection. The results showed that the peak in uNGAL occurred in approximate 1 day, while sCr occurred in approximate 3 days, which implied that the changes in uNGAL were much earlier than those of blood sCr. This indicated that the levels of urinary NGAL may be useful biomarker for predicting renal prognosis of AKI.

Notwithstanding, BOLD MRI can monitor renal oxygenation level affected by CMs administration; however, recent (pre-) clinical studies received the question as to the limitations of renal BOLD MRI [47]. In Seeliger et al.'s study, compared with baseline levels, cortical T2^*∗*^ dropped slightly by about 10% and cortical pO2 reduced by about 40% [48]. The discrepancy between T2^*∗*^ and pO2 response to hypoxia is partially due to the decrease of vascular volume fraction. One reason is that CM-induced high tubular fluid viscosity results in an increase in the intratubular pressure. Another major cause is the vasoconstriction after the administration of CM. Additionally, CM can leftward shift the oxyhaemoglobin dissociation curve, so that release of O2 from haemoglobin (Hb) is impeded. Thus, T2^*∗*^ may not accurately reflect blood oxygenation, and we will continue to work on improving the accuracy of the measurement and the quality of images.

## 5. Limitations

Iodixanol was injected at a dose of 4 g iodine/kg body weight in line with previous studies [29, 49]. The applied dose was based on the body surface area (rat : human = 6 : 1) [50], which mimics the dosage used for humans during contrast medium-enhanced CT (0.5–0.8 g I/kg BW). However, there are a few limitations to be mentioned. First of all, a single dose of CM was applied for each injection. Further research can explore how differences in dosage of repetitive iodixanol will affect the renal functions. Secondly, other risk factors for CIAKI were not considered, such as diabetic nephropathy or chronic renal insufficiency. Additionally, iodixanol was selected as a representative of CMs applied in our study, while further study can compare different CMs with respect to different time intervals. It will be valuable to observe renal damage and to characterize the cause of hypoxia after multiple injections of CMs.

## 6. Conclusions

In conclusion, BOLD and DWI MRI can be applied to examine CIAKI in clinical practice. HIF-1*α* and BOLD imaging can verify the hypoxia tissues from different perspectives in CIAKI. Repetitive injection of iodixanol within a short period of time not only induced more pronounced and longer-lasting renal hypoxia in the whole kidneys, but also enhanced the structure and functions of kidneys as well as elevated urinary biomarkers.

## Figures and Tables

**Table 1:** fMRI parameters applied in BOLD and DWI acquisitions.

	BOLD	DWI
Number of slices	3	3
Section thickness, mm	2.4	2.4
Repetition time, ms	131.5	4500
Echo time, ms	5.4–11	106.9
Orientation	Coronal	Coronal
Bandwidth, hertz per pixel	41.67	62.5–250
Field of view, mm	100 × 100	100 × 100
Matrix	256 × 256	160 × 160
Number of excitations	2	8
Acquisition time	2 min 5 s	1 min 57 s
*b*-values (s/mm^2^)	–	0, 600
Flip angle	30°	90°
Breathing protocol	Free breathing	Free breathing

**Table 2:** R2^*∗*^ values changes in 4 renal regions in all groups over time (s^−1^).

Kidney tissue	Treatment	Baseline (*n* = 17)	1 h (*n* = 17)	1 d (*n* = 14)	3 d (*n* = 11)	5 d (*n* = 8)	10 d (*n* = 5)
CO	1-day subgroup	29.95 ± 2.01	35.75 ± 5.70^*∗*^	35.00 ± 5.82^*∗*^	34.56 ± 5.38^*∗*^	33.70 ± 3.88^*∗*^	32.48 ± 1.61
	3-day subgroup	29.48 ± 1.64	33.75 ± 4.70^*∗*^	33.14 ± 4.28^*∗*^	32.86 ± 4.18^*∗*^	32.24 ± 2.58^*∗*^	30.83 ± 4.20
	5-day subgroup	28.55 ± 3.21	33.25 ± 4.91^*∗*^	32.85 ± 5.17^*∗*^	32.23 ± 3.42^*∗*^	31.36 ± 3.10	29.63 ± 3.41
	Group 1	30.03 ± 1.82	33.95 ± 4.66^*∗*^	33.03 ± 4.07^*∗*^	32.31 ± 3.18^*∗*^	31.05 ± 1.03	30.40 ± 1.38
	Control group	30.36 ± 2.31	29.79 ± 3.61	30.69 ± 2. 72	29.36 ± 3.94	30.83 ± 2.20	29.65 ± 6.07

OSOM	1-day subgroup	33.53 ± 3.06	38.72 ± 4.87^*∗*^	38.12 ± 4.50^*∗*^	37.04 ± 3.02^*∗*^	35.95 ± 2.91	34.57 ± 3.62
	3-day subgroup	33.14 ± 2.36	37.94 ± 6.22^*∗*^	36.79 ± 3.12^*∗*^	36.11 ± 1.63^*∗*^	35.14 ± 4.78	33.50 ± 5.77
	5-day subgroup	31.86 ± 4.40	36.09 ± 1.80^*∗*^	35.23 ± 1.90^*∗*^	33.17 ± 4.21	32.41 ± 1.47	31.94 ± 4.25
	Group 1	34.28 ± 2.69	37.04 ± 3.20^*∗*^	36.31 ± 2.17^*∗*^	35.02 ± 4.75	34.48 ± 2.06	33.63 ± 4.24
	Control group	31.72 ± 5.62	32.30 ± 2.48	32.97 ± 3.50	32.36 ± 2.65	32.54 ± 1.94	33.25 ± 2.46

ISOM	1-day subgroup	35.39 ± 3.55	40.83 ± 4.70^*∗*†^	39.05 ± 5.18^*∗*^	38.02 ± 2.21^*∗*^	37.87 ± 1.09^*∗*^	35.68 ± 4.95
	3-day subgroup	34.99 ± 2.59	40.18 ± 1.43^*∗*†^	38.17 ± 4.00^*∗*^	37.33 ± 1.80^*∗*^	36.73 ± 1.10^*∗*^	34.68 ± 3.58
	5-day subgroup	33.19 ± 1.89	37.68 ± 4.27^*∗*^	36.90 ± 4.22^*∗*^	36.14 ± 3.53^*∗*^	35.13 ± 2.99	34.10 ± 2.20
	Group 1	35.24 ± 1.63	37.78 ± 1.47^*∗*^	37.40 ± 2.89^*∗*^	37.02 ± 2.68^*∗*^	36.29 ± 2.75	34.57 ± 1.81
	Control group	33.60 ± 2.79	33.11 ± 3.82	34.25 ± 4.12	34.32 ± 3.91	34.05 ± 4.77	35.07 ± 1.80

IM	1-day subgroup	29.59 ± 3.60	34.08 ± 5.64^*∗*^	32.99 ± 3.23^*∗*^	32.08 ± 2.84	31.52 ± 2.44	30.72 ± 4.40
	3-day subgroup	29.23 ± 3.87	33.66 ± 5.09^*∗*^	32.15 ± 4.19	31.59 ± 3.81	31.24 ± 5.77	30.03 ± 2.65
	5-day subgroup	28.34 ± 3.62	33.07 ± 4.69^*∗*^	32.01 ± 6.36	31.72 ± 5.54	30.32 ± 2.18	29.25 ± 3.00
	Group 1	28.53 ± 3.13	33.34 ± 4.45^*∗*^	30.96 ± 3.73	30.17 ± 2.66	29.68 ± 1.35	28.14 ± 2.57
	Control group	29.27 ± 3.24	28.87 ± 2.23	29.45 ± 3.60	28.69 ± 2.66	29.61 ± 2.75	28.49 ± 2.21

^*∗*^ *P* < 0.05 versus baseline; ^†^*P* < 0.05 versus Group 1.

**Table 3:** ADC values changes in the 4 renal regions in Group 2 (×10^−5^ mm^2^/s).

Kidney tissue	Treatment	Baseline (*n* = 17)	1 h (*n* = 17)	1 d (*n* = 14)	3 d (*n* = 11)	5 d (*n* = 8)	10 d (*n* = 5)
CO	1-day subgroup	235 ± 10	195 ± 14^*∗*^	214 ± 17^*∗*^	222 ± 10^*∗*^	225 ± 12	229 ± 8
	3-day subgroup	236 ± 8	201 ± 17^*∗*^	217 ± 15^*∗*^	225 ± 13^*∗*^	230 ± 14	232 ± 11
	5-day subgroup	237 ± 8	204 ± 12^*∗*^	221 ± 15^*∗*^	231 ± 8	233 ± 9	240 ± 6

OSOM	1-day subgroup	229 ± 8	187 ± 11^*∗*^	216 ± 17^*∗*^	221 ± 11^*∗*^	227 ± 10	233 ± 7
	3-day subgroup	231 ± 9	196 ± 11^*∗*^	222 ± 14	225 ± 7	232 ± 5	233 ± 5
	5-day subgroup	232 ± 11	206 ± 18^*∗*^	222 ± 18	226 ± 16	229 ± 15	231 ± 6

ISOM	1-day subgroup	208 ± 9	171 ± 17^*∗*^	187 ± 19^*∗*^	191 ± 19^*∗*^	198 ± 18^*∗*^	202 ± 5
	3-day subgroup	206 ± 10	178 ± 12^*∗*^	191 ± 16^*∗*^	201 ± 12	206 ± 11	212 ± 6
	5-day subgroup	208 ± 8	183 ± 17^*∗*^	195 ± 13^*∗*^	203 ± 12	208 ± 11	210 ± 10

IM	1-day subgroup	238 ± 9	166 ± 11^*∗*^	227 ± 19	230 ± 14	234 ± 6	236 ± 10
	3-day subgroup	236 ± 7	171 ± 13^*∗*^	229 ± 13	230 ± 15	236 ± 5	236 ± 6
	5-day subgroup	238 ± 8	182 ± 15^*∗*^	230 ± 15	234 ± 6	236 ± 4	237 ± 9

DWI measurements were performed at baseline and 1 h, 1 day, 3 days, 5 days, and 10 days after the second injection, and mean ADC of each region of interest was measured. ^*∗*^*P* < 0.05 versus baseline.

**Table 4:** The time course of serum creatinine (umol/L) in all the iodixanol-treated groups.

Groups	Baseline (*n* = 3)	1 h (*n* = 3)	1 d (*n* = 3)	3 d (*n* = 3)	5 d (*n* = 3)	10 d (*n* = 3)
1-day subgroup	28.43 ± 3.12	31.97 ± 3.58	35.01 ± 4.38	43.18 ± 3.24^*∗*^	37.26 ± 2.31	32.85 ± 5.04
3-day subgroup	27.59 ± 2.49	30.47 ± 3.53	33.08 ± 5.27	39.12 ± 2.46^*∗*^	34.41 ± 4.08	28.22 ± 2.04
5-day subgroup	28.12 ± 3.29	30.09 ± 3.93	34.24 ± 3.00	36.13 ± 3.73	34.50 ± 2.11	27.46 ± 2.30
Group 1	27.82 ± 3.54	29.33 ± 3.18	32.05 ± 1.68	33.96 ± 2.07	30.02 ± 3.68	27.59 ± 2.67

^*∗*^ *P* < 0.05 versus baseline.
